# The Role of MYCN in Symmetric vs. Asymmetric Cell Division of Human Neuroblastoma Cells

**DOI:** 10.3389/fonc.2020.570815

**Published:** 2020-10-21

**Authors:** Hideki Izumi, Yasuhiko Kaneko, Akira Nakagawara

**Affiliations:** ^1^Laboratory of Molecular Medicine, Life Sciences Institute, Saga-Ken Medical Centre Koseikan, Saga, Japan; ^2^Research Institute for Clinical Oncology, Saitama Cancer Center, Saitama, Japan; ^3^SAGA HIMAT, Tosu, Japan

**Keywords:** MYCN, TRIM32, NCYM, ALDH18A1, asymmetric cell division, neuroblastoma

## Abstract

Asymmetric cell division (ACD) is an important physiological event in the development of various organisms and maintenance of tissue homeostasis. ACD produces two different cells in a single cell division: a stem/progenitor cell and differentiated cell. Although the balance between self-renewal and differentiation is precisely controlled, disruptions to ACD and/or enhancements in the self-renewal division (symmetric cell division: SCD) of stem cells resulted in the formation of tumors in *Drosophila* neuroblasts. ACD is now regarded as one of the characteristics of human cancer stem cells, and is a driving force for cancer cell heterogeneity. We recently reported that MYCN controls the balance between SCD and ACD in human neuroblastoma cells. In this mini-review, we discuss the mechanisms underlying MYCN-mediated cell division fate.

## Introduction

Neuroblastoma is a common cancer in children and exhibits a broad clinical behavior (1–3). Patients are classified into low-, intermediate-, and high-risk groups based on clinical and biological characteristics (1–3). Minimal treatment may be sufficient for the low-risk group, whereas despite intensive treatment, high-risk patients still present with a dismal outcome. The reasons for this heterogeneity remained unclear until molecular, genetic, and biochemical analyses of tumors provided insights into their different clinical behaviors. Among the many genetic and biochemical features of neuroblastoma, amplification of the *MYCN* oncogene correlates with an aggressive phenotype and poor prognosis (1–3). Approximately 20% of neuroblastomas show *MYCN* gene amplification. Recent studies reported that MYCN not only exhibited oncogenic activity, but also played a central role in normal neural stem and progenitor cell self-renewal (4–6).

Neuroblastoma originates from cells of the neural crest, which is a multipotent cell population comprising the embryonic structure (7). The neural crest is composed of migrating cell populations that give rise to diverse cell lineages, including Schwann cells, melanocytes, craniofacial cartilage and bones, smooth muscle, peripheral and intestinal neurons, and glia. Thus, the neural crest acts as pluripotent stem cells that differentiate into mature peripheral nerve tissue. The pluripotent neural crest is suspected to be involved in the tumorigenesis of neuroblastoma due to the abnormal expression of *MYCN*. Neuroblastoma cells are derived from the pluripotent neural crest that has cancer stem cell-like properties (8). Therefore, human neuroblastoma cultured cells exhibit both proliferative and differentiating abilities, and possess similar characteristics to cancer stem cells (9, 10).

Cancer stem cells are considered to undergo asymmetric cell division (ACD), a physiological event resulting in tumor cell heterogeneity (11, 12). ACD is a strategy that maintains the correct number of self-renewing stem cells and differentiated cells in a single division. Therefore, ACD balances the stem cell pool with its progenitor pool. Recent studies revealed that the misregulation of this balance between self-renewal and differentiation by ACD led to the emergence of abnormal stem cells, resulting in tumorigenesis in *Drosophila* neuroblast populations (13). Therefore, cancer stem cells may use ACD as a strategy to generate more cancer stem cells in addition to many differentiated cancer cells. We herein investigated the mechanisms underlying ACD using a series of human neuroblastoma cultured cells as a model system (14–16).

## MYCN

**FIGURE 1:**
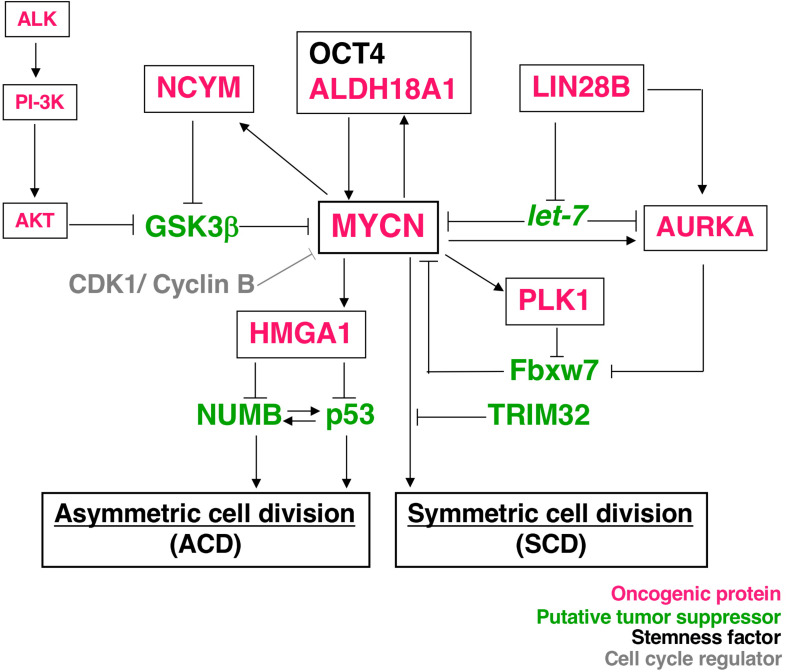
Molecular pathways of MYCN-mediated cell division fate. MYCN protein stability depends on the interaction partners. The receptor type-tyrosine kinase, ALK, activates downstream targets, such as PI-3K, and eventually activates AKT. Activated AKT stabilizes the MYCN protein by inhibiting GSK-3β. NCYM also stabilizes MYCN for inhibition of the GSK3β-MYCN interaction. MYCN induces the expression of NCYM and HMGA1. OCT4 and MYCN or ALDH18A1 and MYCN form a positive feedback loop for their transcriptional expression. HMGA1 inhibits the expression of NUMB and p53. On the other hand, LIN28B inhibits the microRNA, *let-7*, and contributes to the stability of the MYCN and AURKA (Aurora-A) protein. AURKA and PLK1 also stabilize the MYCN protein to inhibit Fbxw7-dependent MYCN ubiquitination. Thus, many oncogenic proteins contribute to the stability of MYCN. As a result, MYCN-dependent tumor cells display symmetric cell division (SCD) and the degradation of MYCN causes asymmetric cell division (ACD).

Asymmetric cell division studies were originally conducted using model organisms, such as nematode embryos (17, 18), *Drosophila* neuroblasts (13), and *Drosophila* germ stem cells (19). The findings of these genetic studies revealed that the mechanism of ACD is highly conserved. Previous studies demonstrated the ACD of stem cells in muscle (20), skin (21), the gut (22), mammary glands (23), the hematopoietic system (24), and the developing mouse brain (25, 26). Comparisons of ACD studies using these organisms and model systems revealed ACD in neuroblastoma cells in an evolutionarily conserved manner (14). The magnitude of *MYCN* gene expression influences the regulation of cell division fate. The overexpression of *MYCN* induces symmetric cell division (SCD) (self-renewal division), and the decreased expression of *MYCN* causes ACD (14). Furthermore, the transcriptional activity of MYCN is important for inducing SCD in human neuroblastoma cells (14). Although the specific transcriptional target(s) of MYCN currently remain unclear, except the high mobility group A1 (HMGA1) oncogene, several key molecular pathways involved in MYCN-mediated cell division fate have been identified (Figure 1).

## TRIM32

**FIGURE 2:**
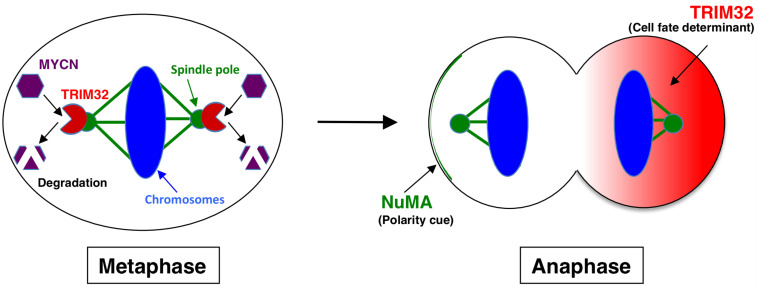
Molecular mechanism underlying TRIM32-mediated asymmetric cell division. During metaphase, TRIM32 is recruited to spindle poles (mitotic centrosomes) by CDK1/cyclin B signaling. In parallel, MYCN accumulates at spindle poles through GSK-3β signaling. Thus, TRIM32 associates with MYCN at spindle poles during metaphase. TRIM32 then facilitates the ubiquitination and degradation of MYCN by a proteasome complex at spindle poles. During anaphase, TRIM32 asymmetrically localizes to the one of the daughter cells without the NuMA crescent.

Tripartite motif-containing 32 (TRIM32) was identified as an ACD inducer in human neuroblastoma cells (15). Previous studies established *TRIM32*, an ortholog of *Drosophila melanogaster*, *Brat*, which participates in ACD as a neural determinant and inhibits *Drosophila* MYC (dMYC) function in the neuroblasts of fly (27). In addition, mouse TRIM32 was shown to exhibit ubiquitin ligase activity, and facilitated the degradation of the c-MYC oncoprotein in neurogenesis (28). However, the functions of TRIM32 in human cancers remain largely unknown. We recently reported that TRIM32 promoted the proteasomal degradation of MYCN at spindle poles during cell division, while *TRIM32* overexpression induced ACD in human neuroblastoma cells (Figures 1, 2) (15). *TRIM3*, another ortholog of *D. melanogaster*, *Brat*, is frequently deleted in human glioblastoma (29). Moreover, TRIM3 has been shown to facilitate the degradation of c-MYC and regulate ACD in human glioma cells (29). Thus, TRIM32/TRIM3 may not only induce ACD, but also function as a tumor suppressor in human tumors.

## NCYM (MYCNOS)

**FIGURE 3:**
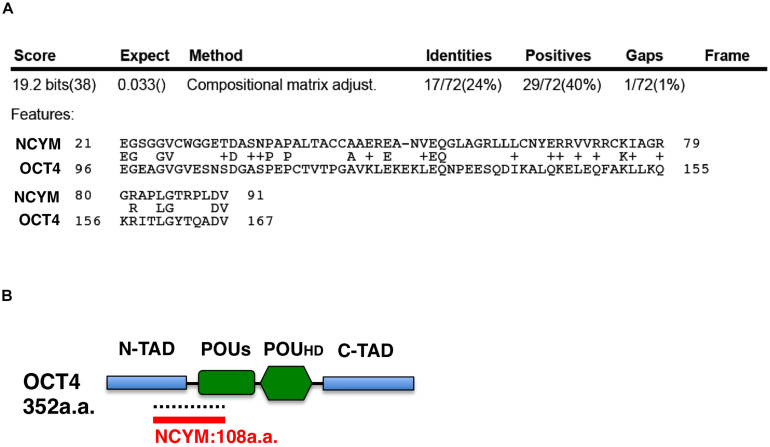
The NCYM protein has weak homology with the OCT4 protein. **(A)** Amino acid comparisons between the NCYM and OCT4 proteins. Identities are 24% and positives are 40%. **(B)** Molecular structure of the OCT4 protein. The OCT4 protein consists of four domains: the N-terminal transactivation domain (N-TAD), C-terminal transactivation domain (C-TAD), POU transcription factor-specific domain (POUs), and POU transcription factor homeodomain (POUHD). OCT4 shares weak homology with NCYM around the N-TAD and POUs domains.

*NCYM* (*MYCNOS*) is a *de novo* evolutionary *cis*-antisense gene for *MYCN* that encodes a 109-amino acid small protein and only exists in humans and chimpanzees (30). NCYM induces the expression of not only MYCN, LIN28B, NANOG, and SOX2, but also OCT4, a MYCN-mediated core reprogramming factor (31). MYCN and OCT4 form a positive feedback loop (Figure 1) (31). A previous study reported that *NCYM* promoted malignant transformation and metastasis in *NCYM/MYCN* double transgenic mice (30). These findings indicated that MYCN cooperates with NCYM to promote the malignant transformation of neuroblastoma and its stemness. NCYM was shown to suppress the degradative activity of GSK3β against MYCN and facilitated the induction of SCD, while the knockdown of *NCYM* destabilized the MYCN protein and caused ACD (Figure 1) (31). On the other hand, *NCYM* also functions as a non-coding RNA and cooperates with CTCF to promote the progression of neuroblastoma by facilitating the expression of *MYCN* (32). Since the NCYM protein has some homology with the OCT4 protein (Figure 3), NCYM may function as a transcription factor in addition to non-coding RNA.

## ALDH18A1

Aldehyde dehydrogenase family 18 member A1 (ALDH18A1) was originally identified as the key enzyme for the synthesis of proline from glutamate, which catalyzes the coupled phosphorylation and reduction conversion of glutamate to β-pyrroline-5-carboxylate (P5C) and plays a critical role in regulating glutamine metabolism (33). A recent study revealed that ALDH18A1 formed a positive feedback loop with MYCN and was involved in the malignant transformation of neuroblastoma cells (Figure 1) (34). These findings demonstrated that the overexpression of *ALDH18A1* decreased the rate of ACD and induced SCD, whereas the knockdown of *ALDH18A1* increased the rate of ACD (34). Furthermore, molecular docking was applied to screen ALDH18A1 inhibitors, and the findings obtained showed that one compound, termed YG1702, from the approximately >200,000 compounds tested specifically inhibited the function of ALDH18A1 (34). Therefore, YG1702 has potential as a therapeutic drug that induces ACD and reduces the malignant transformation of *MYCN*-amplified neuroblastoma cells.

## HMGA1, NUMB, and p53

The important transcriptional target of MYCN in neuroblastoma is the high mobility group A1 (HMGA1) oncogene (35) (Figure 1). The HMGA1 protein is an architectural chromatin protein that is abundantly expressed during embryonic development and in most cancer tissues, but is weakly expressed or absent in normal adult tissues. It is important as an additional potential mechanism by which MYCN may induce the SCD of neuroblastoma stem cells. HMGA1 has been shown to induce the SCD of cancer stem cells by negatively regulating the expression of NUMB (36) or p53 (37) (Figure 1). NUMB is a cell fate determinant, and its expression is the basis for achieving the ACD of stem cells and its expression is either lost or reduced in many tumors (38). In *Drosophila* neuroblasts, NUMB mutations induce the formation of tumors (13). The NUMB protein contributes to the stabilization of p53 by suppressing the effects of HDM2, which is an E3-ubiquitin ligase (39). The deletion of p53 in mammary stem cells was shown to abolish NUMB asymmetry during cell division (23). These findings indicate that p53 and NUMB work in concert with ACD; however, the underlying mechanisms are not yet known in neuroblastoma cells.

## AURKA, PLK1, and LIN28B

Mitotic kinases, such as Aurora kinase A (AURKA) and Polo-like kinase 1 (PLK1), are reported to stabilize MYCN by inhibiting the Fbxw7-mediated degradation of the MYCN protein (40–42) and may promote SCD in *MYCN*-amplified neuroblastoma cells (Figure 1). *AURKA* and *PLK1* are up-regulated by MYCN and are frequently overexpressed in *MYCN*-amplified neuroblastomas (40, 43). On the other hand, *AURKA* and *Polo* (*Drosophila* ortholog of PLK1) were shown to be necessary for asymmetric protein localization during mitosis in model organisms, such as a *Drosophila* external sensory organ (44), and functioned as tumor suppressors in *Drosophila* neuroblasts (45, 46). Therefore, AURKA- and PLK1-mediated cell division fates (ACD or SCD) may be context-dependent.

The *LIN28B* gene encodes a developmentally regulated RNA binding protein and is a key repressor of the *let-7* family of miRNAs, which act as potent tumor suppressors by post-transcriptionally repressing multiple oncogenic targets, including *MYCN* (Figure 1) (47). In neuroblastoma cells, LIN28B promotes AURKA expression (48) and increases MYCN expression by repressing *let-7* miRNAs (47). Since LIN28B is involved in SCD in *Drosophila* intestine stem cells (49) and *Caenorhabditis elegans* embryos (50), it may control the cell division fate in neuroblastoma cells in an evolutionarily conserved manner.

## Concluding Remarks

Why do neuroblastoma cultured cells exhibit ACD? As discussed above, many cultured neuroblastoma cells show the unique characteristics of proliferation and differentiation capabilities (9, 10). Previous studies demonstrated that human neuroblastoma cell lines may be classified into three distinct cellular phenotypes with different differentiation potentials: the sympathoadrenal neuroblast type (N-type), substrate adherent type (S-type), and intermediate type (I-type) (9, 10). Based on its morphological and growth characteristics, the I-type was recognized as a neuroblastoma stem cell by its unique differentiation and malignant potentials (9, 10). In addition, neuroblastoma is a typical childhood cancer that may arise at the fetal development stage when a large number of stem cells exhibit ACD.

In this mini-review, we discussed a group of molecules that are involved in ACD and SCD through the regulation of the MYCN protein. Since the major function of MYCN is as a transcription factor, further studies are needed to clarify whether HMGA1, a target of MYCN, is involved in the cell division fate of human neuroblastoma cells.

Since this mini-review mainly described the intrinsic factors of ACD, further studies are needed on extrinsic factors including the tumor microenvironment. The findings obtained may contribute to direct applications for therapeutic strategies.

Therefore, human neuroblastoma cultured cells have potential as a very useful model system for providing insights into the mechanisms underlying ACD.

## Author Contributions

HI performed a literature review and submitted the manuscript to the journal. HI, YK, and AN wrote the manuscript. All authors contributed to the article and approved the submitted version.

## Conflict of Interest

The authors declare that the research was conducted in the absence of any commercial or financial relationships that could be construed as a potential conflict of interest.

## Abbreviations

ACD: asymmetric cell division
ALDH18A1: Aldehyde dehydrogenase family 18 member A1
ALK: Anaplastic lymphoma kinase
AURKA: Aurora kinase A
Brat: brain tumor
CDK1: cyclin dependent kinase 1
C-TAD: C-terminal transactivation domain
CTCF: CCCTC-binding factor
Fbxw7: F-box and WD repeat domain-containing 7
GSK3β: glycogen synthase kinase 3 β
HMGA1: high mobility group A1
MYCNOS: MYCN opposite strand
N-TAD: N-terminal transactivation domain
NuMA: Nuclear mitotic apparatus
OCT4: Octamer-binding transcription factor 4
PI-3K: Phosphoinositide 3-kinase
PLK1: Polo-like kinase 1
POUHD: POU transcription factor homeodomain
POUs: POU transcription factor-specific domain
SCD: symmetric cell division
TRIM3: tripartite motif-containing 3
TRIM32: tripartite motif-containing 32.
